# Tratamento da recidiva da crossa da veia safena magna por acesso proximal

**DOI:** 10.1590/1677-5449.004217

**Published:** 2017

**Authors:** Paula Dayana Matkovski, Jorge Oliveira da Rocha, Arthur de Souza Rocha, André Laurindo Cabral, Caio Augusto Knihs, João Marcelo Gonçalves da Rocha Loures, Fabrício Martins Zucco, Patrick Cardoso Candemil

**Affiliations:** 1 Pontifícia Universidade Católica do Paraná – PUC-PR, Departamento de Ciências da Saúde, Curitiba, PR, Brasil.; 2 Hospital Santa Isabel – HSI, Setor de CirurgiaVascular, Blumenau, SC, Brasil.; 3 Fundação Educacional Serra dos Órgãos – UNIFESO, Centro de Ciências da Saúde, Teresópolis, RJ, Brasil.

**Keywords:** veia safena, recidiva, varizes

## Abstract

A maior causa de recidiva das varizes dos membros inferiores é a inadequada dissecção da crossa da safena interna, com a ligadura não rente da junção safenofemoral. O acesso direto ao tecido cicatricial de uma cirurgia prévia deve ser evitado ao máximo pelo elevado risco de sangramento e de lesões linfáticas. O acesso proximal ao tecido cicatricial, abordando inicialmente a veia femoral comum acima da junção safenofemoral, seguindo-a em direção caudal até a crossa da safena, mostrou-se uma técnica eficiente e relativamente simples quando comparada às abordagens medial, lateral e direta.

## INTRODUÇÃO

A maior causa de recidiva das varizes dos membros inferiores é a inadequada dissecção da crossa da safena interna, com a ligadura não rente da junção safenofemoral^1^^,^ 2. A cirurgia para a reexploração dessa região é frequentemente difícil devido ao tecido cicatricial, envolvendo as delicadas e dilatadas veias varicosas dessa localização^3^. O acesso direto sobre o tecido cicatricial deve ser evitado ao máximo pelo elevado risco de sangramento e de lesões linfáticas.

Entre alguns cirurgiões vasculares, o acesso lateral, com visualização inicial da artéria femoral, tem sido uma via natural para a correção desse problema^1^^,^ 4. Outros cirurgiões, como Dodd e Cockett^5^, sugerem o acesso medial como sendo a melhor via para essa abordagem. Em contrapartida, o acesso proximal ao tecido cicatricial, descrito inicialmente por Luke^6^ e Lofgren et al.^7^, abordando inicialmente a veia femoral comum, acima da junção safeno-femoral, seguindo-a em direção caudal até a crossa da safena é considerada por nós uma técnica simples e rápida para a abordagem da recidiva da crossa da safena. Atualmente, o acesso proximal é também utilizado pelos cirurgiões vasculares para a exposição dos vasos femorais durante o implante das endopróteses de aorta.

### Descrição do caso

No período de março de 2012 a dezembro de 2016, 62 pacientes consecutivos, não selecionados, com queixa de recorrência de varizes em membros inferiores e com aparente ligadura prévia da junção safenofemoral, foram diagnosticados com recidiva da crossa da safena interna. Foram abordados 74 membros inferiores, sendo 23 direitos, 27 esquerdos e 12 bilaterais. Em todos os casos, utilizou-se o estudo dúplex venoso para confirmação da recidiva da crossa.

Todos os pacientes foram operados em centro cirúrgico por técnica asséptica, com bloqueio raquimedular, e receberam alta hospitalar após 7 horas de internação. Após a alta, todos foram estimulados a deambular e utilizar elastocompressão.

**Figura 1:**
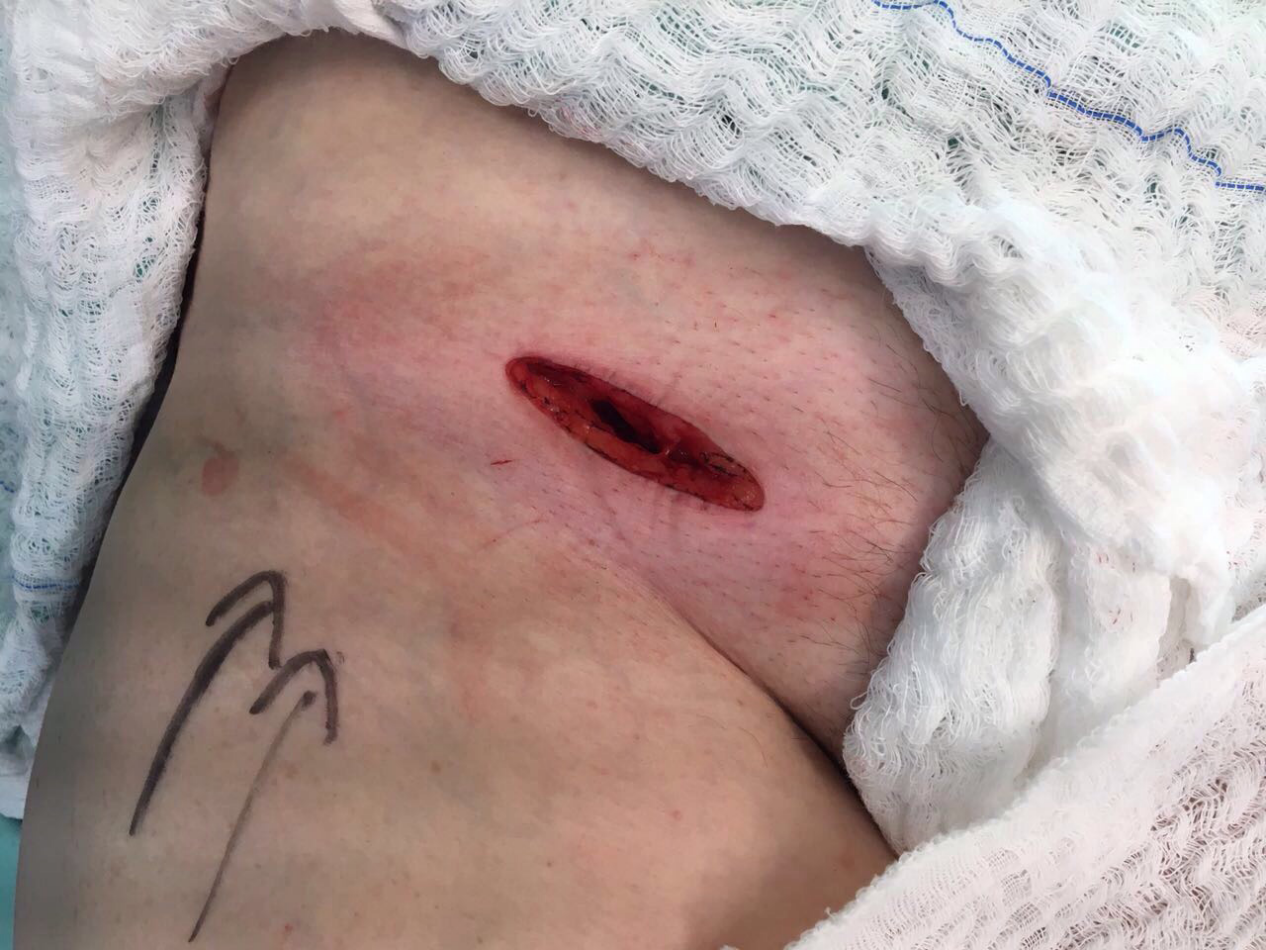
Incisão oblíqua acima do ligamento longitudinal.

**Figura 2:**
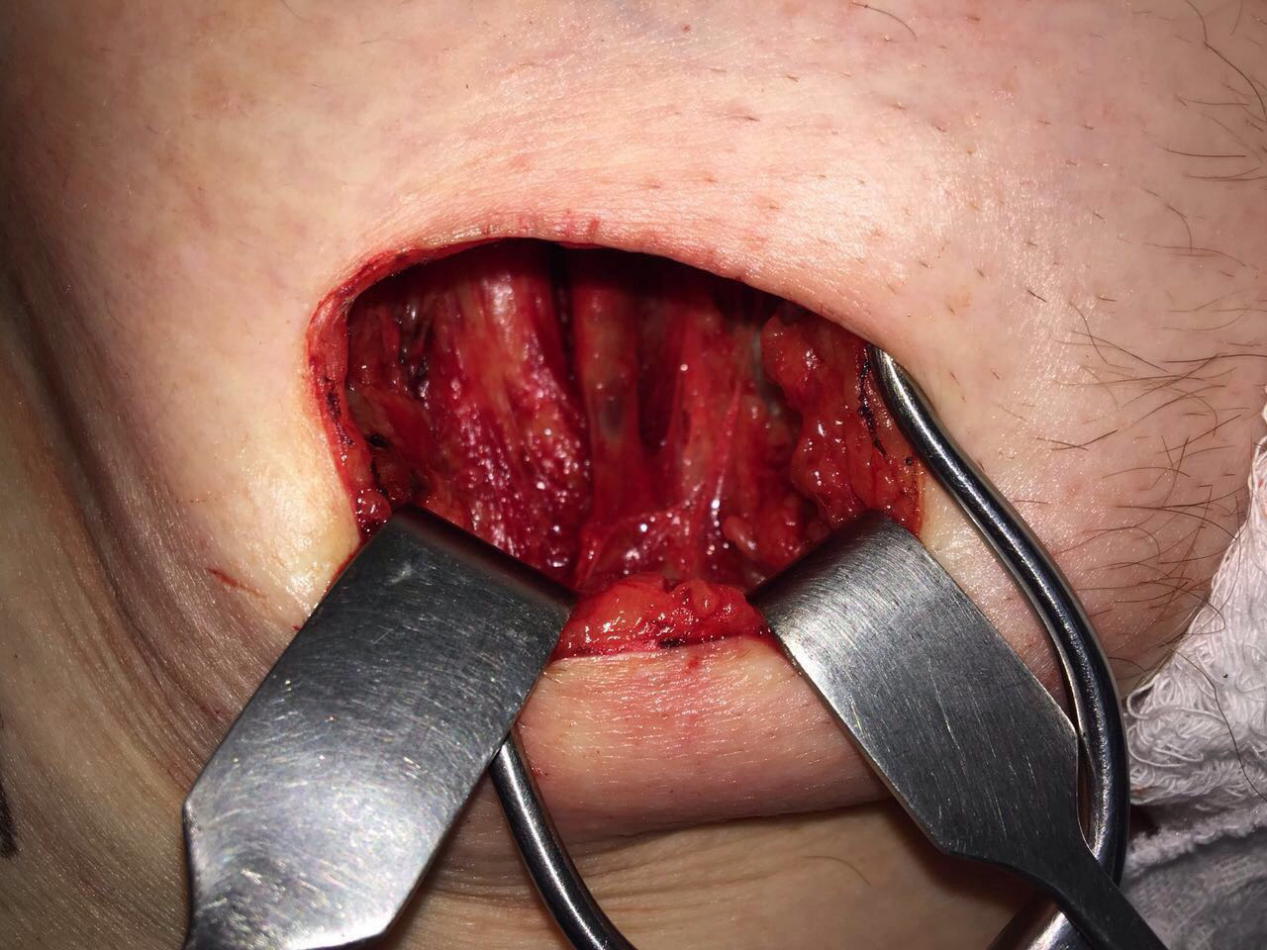
Identificação da crossa da safena e da veia femoral comum.

**Figura 3:**
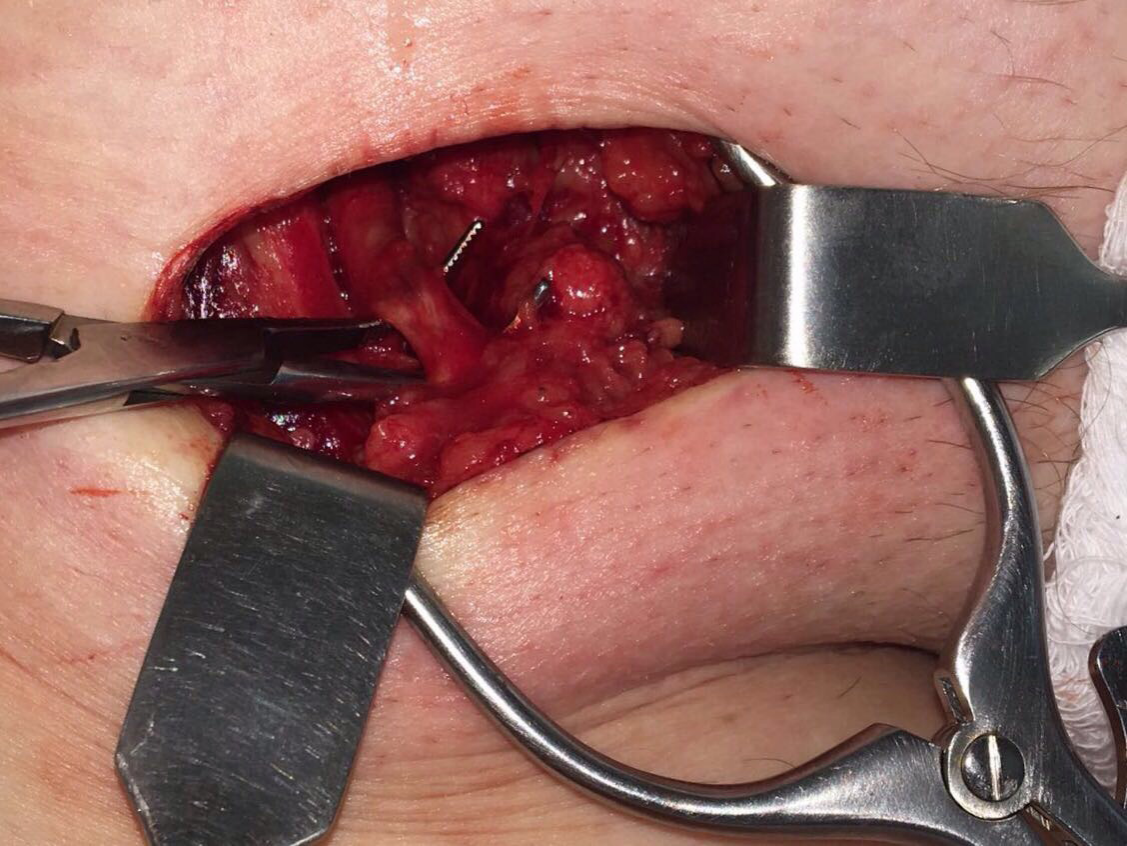
Isolamento da crossa da safena.

Com relação à técnica de reexploração da crossa por abordagem proximal, realizou-se a palpação da fossa oval e a identificação do ligamento inguinal, bem como a visualização da cicatriz da incisão da safenectomia prévia. Uma incisão oblíqua de 5-6 cm foi realizada aproximadamente 1 cm acima do ligamento inguinal (Figura 1), sendo aprofundada até a fáscia transversal. Na sequência, foram identificados o ligamento inguinal e uma pequena sombra da veia femoral comum. Em caso de dificuldade na identificação da veia femoral comum, a palpação da artéria femoral comum poderá facilitar a tarefa em situação medial (Figura 2). Seguiu-se com a dissecção longitudinal sobre a região anterior da veia femoral comum por planos avasculares de Leriche, em direção caudal até a completa exposição da junção safenofemoral, bem como das tributárias da veia femoral comum, evitando, assim, danos aos vasos linfáticos. Uma vez que a junção safenofemoral e a veia femoral comum foram identificadas, a crossa da safena interna foi ligada de forma rente à veia femoral comum, com fio inabsorvível, sendo dividida a seguir (Figuras 3 e 4). Para a síntese, foi realizada a aproximação dos *flaps* da fáscia pectínea e do tecido subcutâneo com fio absorvível e com fio monofilamentar não absorvível para a pele.

**Figura 4:**
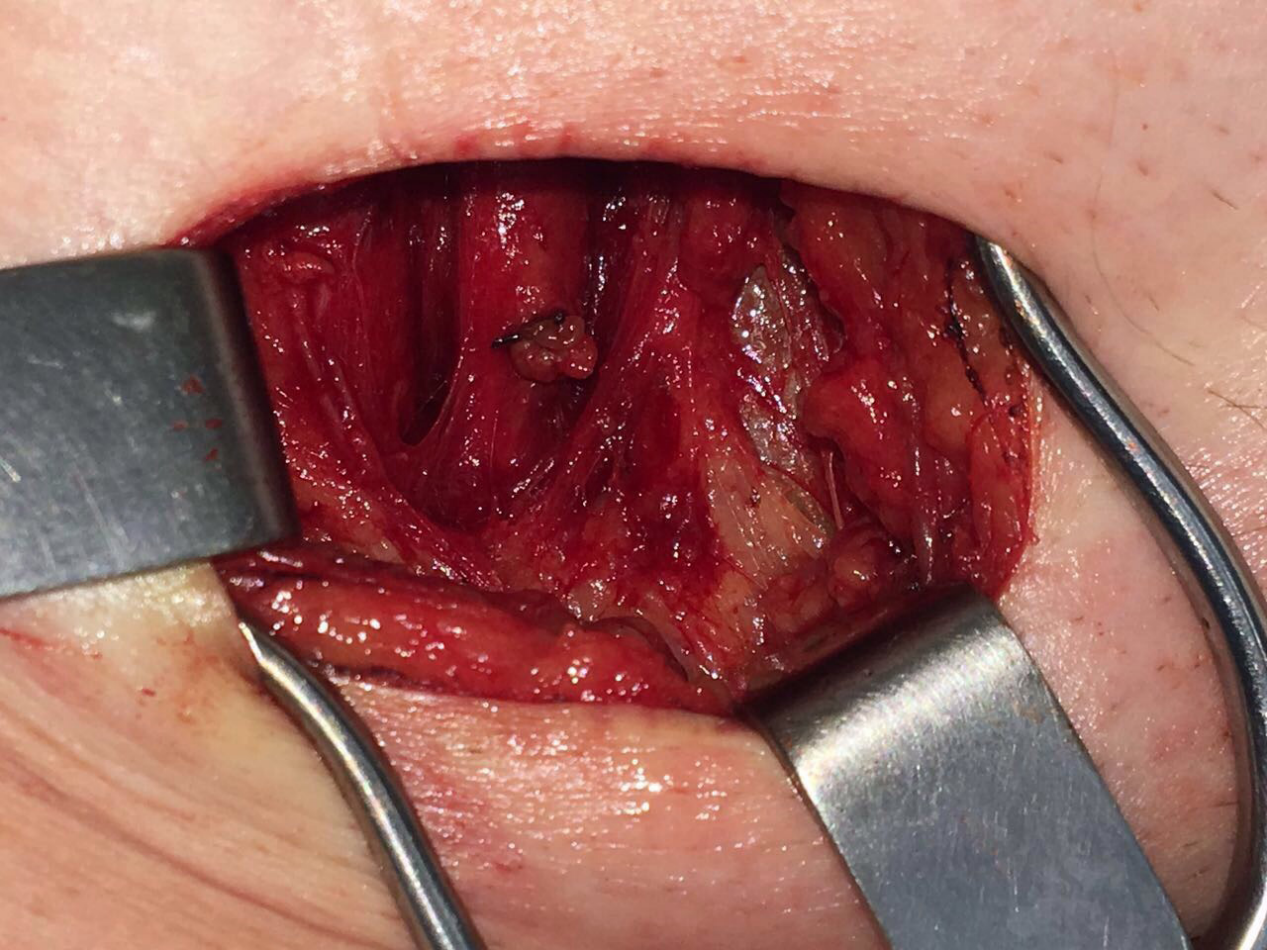
Ligadura da crossa da safena.

## DISCUSSÃO

A recorrência das varizes dos membros inferiores não é incomum. Sheppard^8^ relatou uma taxa de 28% de recorrência de varizes após uma cirurgia prévia, com elevado número associado à incompetência da junção safenofemoral. Embora acreditasse que a ligadura distante da junção safenofemoral fosse a causa mais comum e óbvia da recorrência, Sheppard^8^ também observou recidivas onde foram realizadas ligaduras das junções safenofemorais de forma rente às veias femorais comuns. Esses achados corroboram a opinião de Lofgren et al.^7^, que identificou pequenas conexões das veias, mesmo com a correta ligadura da crossa da safena interna com a veia femoral comum, nas virilhas reexploradas. Qualquer que seja a causa da recorrência da incompetência safenofemoral, a reabordagem será difícil. Diversos autores sugerem evitar o acesso direto à área com tecido cicatricial devido aos riscos inerentes de lesão dos vasos linfáticos e à grande perda sanguínea decorrente da abertura inadvertida das delicadas e abundantes veias varicosas dessa topografia.

Ficamos satisfeitos com a simplicidade do acesso proximal à junção safenofemoral e com os resultados obtidos. Com a abordagem proximal, observamos um menor número de vasos no trajeto até a junção safenofemoral com necessidade de ligadura, quando comparada às abordagens medial, lateral e direta. Deve-se considerar também que raramente os vasos varicosos se desenvolvem em direção cranial, o que torna esse acesso relativamente livre de varizes e/ou tecidos cicatriciais. Em decorrência da dissecção da veia femoral comum longitudinalmente em direção caudal, os vasos linfáticos também apresentam uma relativa proteção.

Como complicações precoces, observamos um caso de infecção da ferida operatória tratada com sucesso e um caso de linfedema discreto com melhora espontânea após 6 meses da cirurgia. Não houve necessidade de hemotransfusão em nenhum dos casos. Uma queixa relativamente frequente dos pacientes submetidos à abordagem da junção safenofemoral por via proximal foi a anestesia discreta da região inguinal, que apresentou melhora espontânea após alguns meses da cirurgia. Atribuímos essas complicações precoces à quebra de protocolos técnicos relativos a higiene e cuidados pós-operatórios pelo paciente, e possivelmente à curva de aprendizado, devido à ausência de novas complicações semelhantes. Com relação à queixa de anestesia/hipossensibilidade que frequentemente é relatada, trata-se de um quadro transitório e autolimitado, sem maiores limitações para o paciente.

Em nosso entendimento, a abordagem cirúrgica para o tratamento da recidiva da crossa da safena interna é a única técnica que pode remover a junção safenofemoral de maneira eficaz. A escleroterapia com espuma densa pode atingir a crossa, mas tem como desvantagens o alto risco de trombose venosa profunda e as altas taxas de recanalização em curto prazo, proibitivas em nossa opinião. Por fim, os métodos de termoablação endoluminais tratam as tributárias remanescentes, mas deixam a junção safenofemoral intacta.
