# Supplementary material for: Tratamento da recidiva da crossa da veia safena magna por acesso proximal
Source: J Vasc Bras. 2017 Oct-Dec;16(4):335–8. [Article in Portuguese] doi: 10.1590/1677-5449.004217 (PMC5944313; doi:10.1590/1677-5449.004217)
Supplement: Translation

# Translation: Treatment of recurrent varicosity of the great saphenous vein arch via proximal access

**Authors:** Paula Dayana Matkovski, Jorge Oliveira da Rocha, Arthur de Souza Rocha, André Laurindo Cabral, Caio Augusto Knihs, João Marcelo Gonçalves da Rocha Loures, Fabrício Martins Zucco, Patrick Cardoso Candemil

**Affiliations:** 1 Pontifícia Universidade Católica do Paraná – PUC-PR, Department of Health Sciences, Curitiba, PR, Brazil.; 2 Hospital Santa Isabel – HSI, Sector of Vascular Surgery, Blumenau, SC, Brazil.; 3 Fundação Educacional Serra dos Órgãos – UNIFESO, Health Sciences Center, Teresópolis, RJ, Brazil.

**Keywords:** saphenous vein, recurrence, varicose veins

## Abstract

The most common cause of recurrence of varicose veins in the lower limbs is inadequate dissection of the internal saphenous arch, with ligature that is not flush to the saphenofemoral junction. Direct access to the scar tissue of previous surgery should be avoided to the maximum because of the high risk of bleeding and of lymphatic injuries. Access proximal to the scar tissue, initially at the common femoral vein, above the saphenofemoral junction, continuing caudally to the saphenous arch, proved to be an effective and relatively simple technique when compared to medial, lateral and direct approaches.

## INTRODUCTION

The most common cause of recurrence of varicose veins in the lower limbs is inadequate dissection of the internal saphenous arch, with ligature that is not flush to the saphenofemoral junction.^1^^,^ 2 Repeat surgery to explore this area again is often difficult because of scar tissue, involving delicate and dilated varicose veins in this area.^3^ Direct access over the scar tissue should be avoided to the maximum because of the high risk of bleeding and of lymphatic injuries.

Some vascular surgeons have come to consider a lateral access, with the femoral artery revealed first, as the natural route to correct the problem.^1^^,^ 4 Other surgeons, such as Dodd and Cockett,^5^ suggest medial access is the best route to for this intervention. However, we consider that obtaining access proximal to the scar tissue, as first described by Luke^6^ and Lofgren et al.,^7^ initially approaching the common femoral vein above the saphenofemoral junction and following it caudally to the saphenous arch, is a simple and rapid technique for management of recurrent internal saphenous arch varicosity. Nowadays, the proximal access is also used by vascular surgeons to expose femoral vessels during placement of aorta endoprostheses.

### Case description

During the period from March 2012 to December 2016, 62 consecutive unselected patients, with recurrence of varicose veins in the lower limbs and with ligature apparently short of the saphenofemoral junction, were diagnosed with recurrent internal saphenous arch varicosity. A total of 74 lower limbs were treated, 23 right, 27 left and 12 bilateral. In all cases, Duplex venous examination was used to confirm arch recurrence.

All patients were treated in an operating theatre with aseptic techniques and spinal anesthesia and were discharged from hospital after 7 hours. All were instructed to walk after admission and wear elastic compression devices.

The technique used to reintervene at the arch, via proximal access was as follows. Initially, the saphenous opening was palpated, the inguinal ligament identified, and the incision scar from the previous saphenectomy was located. A 5-6 cm oblique incision was made approximately 1 cm above the inguinal ligament and continued to the depth of the transverse fascia (Figure 1). Next, the inguinal ligament and the small shadow of the common femoral vein were identified. In cases in which it proves difficult to identify the common femoral vein, palpation of the common femoral artery at a medial point can facilitate the task (Figure 2). The next step is longitudinal dissection over the anterior common femoral vein by Leriche avascular layers in the distal direction, until the saphenofemoral junction has been completely exposed along with the common femoral vein’s tributaries, thus avoiding damage to lymph vessels. Once the saphenofemoral junction and common femoral vein have been identified, the internal saphenous arch is ligated flush to the common femoral vein, with non-absorbable thread, and then dissected (Figures 3 and 4). For closure, the flaps of the pectineus fascia and the subcutaneous tissue were drawn together with absorbable thread and the skin with non-absorbable monofilament sutures.

**Figure 1:** Oblique incision above the longitudinal ligament.

**Figure 2:** Identification of the saphenous arch and the common femoral vein.

**Figure 3:** Isolation of the saphenous arch.

**Figure 4:** Ligature of the saphenous arch.

## DISCUSSION

Recurrence of varicose veins is not uncommon in the lower limbs. Sheppard^8^ reported a rate of 28% of varicose vein recurrence after prior surgery, with a high number associated with saphenofemoral junction incompetence. Although he believed that ligature distant from the saphenofemoral junction was the most common and obvious cause of recurrence, Sheppard^8^ also observed recurrence in cases in which the saphenofemoral junctions were ligated flush to the common femoral veins. These findings corroborated the opinion of Lofgren et al.,^7^ who had observed small connections to veins in re-operated groins, even when ligature of the internal saphenous arch at the common femoral vein had been performed correctly. Whatever the cause of recurrence of saphenofemoral incompetence, re-operation will be difficult. Many authors suggest that direct access to the area with scar tissue should be avoided because of the inherent risks of injuring lymph vessels and the major blood loss caused by inadvertently opening the delicate and abundant varicose veins in this region.

We have been satisfied by the simplicity of proximal access to the saphenofemoral junction and with the results achieved. Taking the proximal route, we observed a smaller number of vessels along the path to the saphenofemoral junction needing ligature, when compared to the medial, lateral and direct approaches. It should also be considered that varicose vessels will rarely develop in the cranial direction, which means that this route is relatively free from varicose veins and/or scar tissue. Longitudinal dissection of the common femoral vein in the caudal direction also offers a relative degree of protection for the lymph vessels.

With regard to early complications, we observed one case of infection of the surgical wound that was treated successfully and one case of discrete lymphedema with spontaneous resolution 6 months after surgery. There was no need for blood transfusion in any of the cases. One relatively common complaint among patients subjected to saphenofemoral junction intervention via the proximal route was discrete anesthesia of the groin, which resolved spontaneously some months after surgery. We attributed these early complications to failure to follow technical protocols related to hygiene and postoperative care by the patient, and possibly to a learning curve, in view of an absence of similar complications later. With relation to the complaint of anesthesia/hyposensitivity that is frequently reported, this is a transitory and self-limiting condition that does not cause the patient significant limitations.

To our understanding, surgical reintervention to treat recurrent internal saphenous arch varicosity is the only technique that can effectively remove the saphenofemoral junction. Dense foam sclerotherapy may reach the arch, but it has the disadvantages of high risk of deep venous thrombosis and high rates of recanalization over the short term, which in our opinion are prohibitive. Finally, endothermal ablation methods treat remnant tributaries, but leave the saphenofemoral junction intact.
